# Absence of diurnal variation in visceromotor response to colorectal distention in normal Long Evans rats

**DOI:** 10.12688/f1000research.7238.1

**Published:** 2016-01-22

**Authors:** Sara Botschuijver, Zhumei Yu, Olaf Welting, Cathy Cailotto, Andries Kalsbeek, Rene van den Wijngaard

**Affiliations:** 1 Department of Gastroenterology and Hepatology, Tytgat Institute for Liver and Intestinal research, Academic Medical Center, Amsterdam, Amsterdam, Netherlands; 2 Department of Neurobiology, Tongji Medical College, HUST, Wuhan, China; 3 Department of Endocrinology and Metabolism, Academic Medical Center, University of Amsterdam, Amsterdam, Netherlands

**Keywords:** Circadian, clock, diurnal, irritable bowel syndrome, IBS, perception, rat, visceral perception

## Abstract

**Background:** Enhanced colorectal sensitivity (i.e. visceral hypersensitivity) is thought to be a pathophysiological mechanism in irritable bowel syndrome (IBS). In healthy men a circadian variation in rectal perception to colonic distention was described. Disturbed day and night rhythms, which occur in shift work and trans meridian flights, are associated with the prevalence of IBS. This raises the question whether disruptions of circadian control are responsible for the observed pathology in IBS. Prior to investigating altered rhythmicity in relation to visceral hypersensitivity in a rat model for IBS, it is relevant to establish whether normal rats display circadian variation similar to healthy men.

**Methodology and findings:** In rodents colorectal distension leads to reproducible contractions of abdominal musculature. We used quantification of this so called visceromotor response (VMR) by electromyography (EMG) to assess visceral sensitivity in rats. We assessed the VMR in normal male Long Evans rats at different time points of the light/dark cycle. Although a control experiment with male maternal separated rats confirmed that intentionally inflicted (i.e. stress induced) changes in VMR can be detected, normal male Long Evans rats showed no variation in VMR along the light/dark cycle in response to colorectal distension.

**Conclusions:** In the absence of a daily rhythm of colorectal sensitivity in normal control rats it is not possible to investigate possible aberrancies in our rat model for IBS.

## Introduction

Irritable bowel syndrome (IBS) is one of the most common functional gastrointestinal disorders and abdominal pain is the key contributing factor ^1^. Up to 50% of patients have increased perception of gastrointestinal stimuli and this so called visceral hypersensitivity (assessed by rectal balloon distension) is considered a major pathophysiological mechanism ^2^. Circadian variation in perception of rectal distention was described in healthy male volunteers ^3^. The observed rhythmicity may relate to the autonomic nervous system being under the regime of the circadian clock ^4^. This raises the question whether visceral hypersensitivity in IBS can be explained by disrupted circadian control of the autonomic nervous system. A subtle increase in sympathetic and decrease in parasympathetic nervous activity was observed in patients ^5^ and complaints are known to show a daily variation, with higher pain scores early in the morning ^6^. In addition, a disturbed day/night rhythm, which occurs in shift work and after transmeridian flights, is associated with the prevalence of IBS ^7, 8^. We wanted to investigate whether disrupted circadian control of the autonomic nervous system can explain post-stress hypersensitivity to colorectal distension observed in a rat model of IBS (i.e. the well validated maternal separation model ^9, 10^). However, before investigating possible aberrancies it was essential to confirm, in our experimental setting, earlier reported circadian variation of perception in normal ‘non-IBS’ rats ^11^. In rodents, colorectal balloon distension leads to reproducible contractions of abdominal musculature (the visceromotor response [VMR]). The electromyographical (EMG) quantification of this response is often used to assess visceral sensitivity and changes thereof in rodents ^10, 12^. We used VMR quantification by EMG to assess possible circadian variation of visceral perception in Long Evans rats.

## Methods

### Animal ethics

All research was conducted in accordance with the institutional guidelines and approved by the Animal Ethical Committee of the AMC/University of Amsterdam (reference protocol number 100998).

### Rats

25 Long Evans rats (Harlan, Horst, Netherlands) were bred and housed at the animal facility of the Academic Medical Center (Amsterdam, Netherlands). Rats were housed in a 12 h light/12 h dark cycle (lights on at 07:00) under a constant temperature of 20±2C° and were provided with food and water *ad libitum*.

### Measurement of the visceromotor response to colonic distension

To avoid restraint stress during measurements, we previously validated and used radio-telemetry for assessing the VMR in freely moving rats ^12^. The same methodology was used in the current investigations. In short, at a minimum age of 4 months, a telemetric transmitter with its two connected EMG electrodes (Physiotel Implant TA10AE-F20; Data Sciences International, St Paul, MN, USA) was implanted in the right side of the abdominal cavity. The electrodes were sutured in parallel into the left external abdominal oblique muscle 10 mm apart and 10 mm to the midline incision. After a postoperative recovery period of at least 10 days, the animals were subjected to colonic distention protocols. For this purpose, a latex balloon (Ultracover size 8F; International Medical Products BV, Zutphen, Netherlands) catheter was inserted 1 cm into the colon and fixed to the base of the tail under a short isoflurane anesthesia. After a 20 min recovery period, animals were placed in a macrolon cage (exact size of the receiver) that was positioned on top of the receiver. The receiver was linked to a Biopac MP100 data acquisition system (Biopac Systems Inc., Santa Barbara, CA, USA) and a personal computer via a raw data analog converter (Data Sciences International). Data were acquired with AcqKnowledge software (version 3.2.6, Biopac Systems Inc.). Colonic distention was achieved by slow manual inflation (5 s) of graded volumes of water (1.0, 1.5 and 2.0 mL) into the balloon using a syringe. Length and diameter of the balloon during a 2 mL maximum volume distention were 18 and 15 mm, respectively. All distensions lasted for 20 s and were separated by an 80 s rest in order to allow the EMG signal to return to baseline.

### Diurnal measurements of VMR to distension in normal nonhandled rats

Our earlier investigations indicated that repetitive distension sessions in normal male Long Evans rats (carried out at 09:00 on different days during a one month time period) result in equal VMR data ^13^. Thus, inter distension-session interference is ruled out when repetitive measurements are carried out on different days. In the present investigations the VMR to colonic distension of 16 male nonhandled rats was measured at four different time points; 04:00, 08:00, 16:00, and 20:00. Experimental bias was avoided by stratifying rats into 4 equal groups: one group of four rats started sessions at 04:00, followed by 08:00, 16:00 and 20:00, another group started at 08:00 followed by 16:00, 20:00 and 04:00 etc. There was a minimum of 24 hours between distension sessions and all sessions within one group were completed within 15 days. To ascertain proper timing, not more than four animals per session were measured. During dark regime measurements, experiments were carried out under dim red light conditions and the experimental room was devoid of the regular tube light of the housing facility.

### Stress induced visceral hypersensitivity in maternal separated rats

 In adult male maternal separated Long Evans rats, acute stress is known to induce enhanced sensitivity to colorectal distension ^12– 15^. To assure that, when present, our methodology can accurately assess changes in VMR to colorectal distension, comparison of pre- and post-stress measurements in maternal separated rats was used as a positive control. Maternal separation was accomplished by placing the dams into another cage in another room for 180 minutes per day from postnatal day 2 to 14. During separation, cages were placed on a heating pad (30–34ºC) to help pups regulate normal body temperature. Pups were weaned on day 22 and subsequently raised in pairs of two until the age of 4 months when they were subjected to the experimental protocol. Earlier, we showed that post-stress hypersensitivity to distension will last for at least one month ^13^. In line with the multiple distension sessions performed in the diurnal experiment (all four sessions carried out within a maximum timeframe of 15 days), colorectal distensions and concurrent EMG measurements were carried out pre- and 15 days post-stress. Rats were subjected to one single stress session by placing individual rats on top of a pedestal (8 · 8 · 10 cm) attached to the bottom of a plexiglass tank (25 · 25 · 45 cm). The tank was filled with fresh tap water at room temperature (21ºC) within 1 cm of the top of the pedestal and rats remained in the tank for 1 hour.

### Data analysis and statistics

Data analysis was carried out similar to our earlier publications ^12, 14, 15^. Each 20 s distension period and its preceding 20 s of baseline recording were extracted from the original raw EMG data file. After correction for movement and breathing, data were rectified and integrated. Absolute data sets were then obtained by subtracting the 20 s baseline recording from the 20 s distension result. Normalized data sets were then calculated from the absolute data by setting, in case of diurnal measurements, the 2 mL value of the 04:00 distension session, and in case of maternal separated rats the 2 mL value of the pre-stress distension session, at 100%. Area under the curve (AUC) of relative responses was calculated for individual rats and used to show possible changes in visceromotor response within groups. Relative response data were also used to evaluate possible changes on a per volume basis.

Statistical analyses were performed using GraphPad Prism version 5.00 for Windows (GraphPad Software, San Diego, CA, USA). Diurnal variation in VMR (AUC as well as per volume results of the relative response) were evaluated by Friedman’s test. Pre- vs post-water avoidance data (AUC and per volume results of the relative response) of maternal separated rats were analysed by using Wilcoxon signed ranks test. All results are displayed as mean ± SEM and *P*<0.05 was considered significant.

## Results

**Figure 1.**
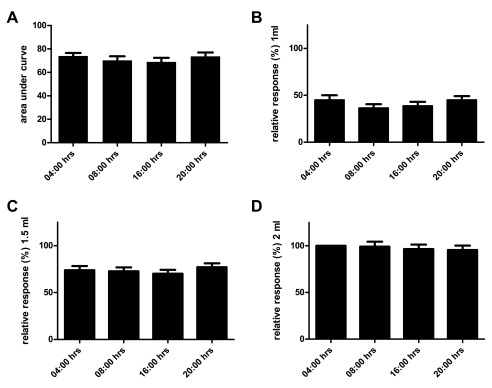
Diurnal pattern of VMR to distension in normal male Long Evans rats. Mean area under the curve ± SEM of the relative responses at 04:00, 08:00, 16:00 and 20:00 hrs in n=16 normal male Long Evans rats (Friedman’s test *P*=0.69) ( **A**). Using the same data set but now depicting per volume relative responses on different time points (Friedman’s test *P*=0.55, 0.95 and 0.93; **B**, **C** and **D** respectively).

**Figure 2.**
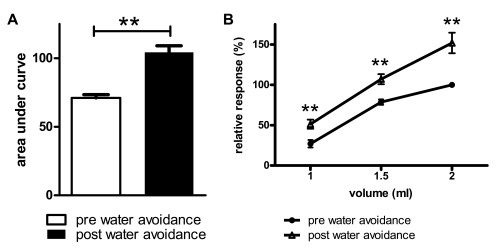
Pre- vs post-water avoidance VMR to distension in maternal separated male Long Evans rats. Pre- vs post-water avoidance area under the curve ± SEM of the relative response to distension ( **A**). Same data set now depicted for per volume responses; increased post-stress response to distension at all 3 distension volumes ( **B**). n=9, Wilcoxon’s test ** *P*<0.01.

Excel datasheet showing raw EMG data acquired during colonic distensions Also shown are the normalized datasets used to compile Figure 1 and Figure 2 depicting diurnal pattern of visceromotor response to distension in normal rats and the control experiment showing maternally separated rats before and after water avoidance stress. Click here for additional data file. Copyright: © 2016 Botschuijver S et al. 2016 Data associated with the article are available under the terms of the Creative Commons Zero "No rights reserved" data waiver (CC0 1.0 Public domain dedication).

### Diurnal VMR to distension in normal male rats

We assessed the VMR to distension in normal male Long Evans rats along four different time points of the light/dark cycle. Due to battery failure of its telemetric transmitter one animal could not be evaluated. Comparing area under the curve (AUC) of the relative response at the four time points we observed no daily variation ( Figure 1A, n=15, Friedman’s test *P*=0.69). Since the AUC reflects the mean visceral sensitivity over a range of distension volumes, more subtle daily variations are perhaps better reflected when comparing results on a per volume basis. However, no significant differences were observed when comparing results for 1.0, 1.5 and 2.0 mL on the different time points ( Figure 1B–D, Friedman’s test *P*=0.55, 0.95 and 0.93 respectively).

### Pre- vs post-water avoidance VMR to distension in maternal separated rats

 We next performed a control experiment to ascertain that, when present, our methodology was capable of detecting changes in VMR to distension. Similar to our earlier publications, male Long Evans pups were subjected to the maternal separation protocol and exposed to a one hour water avoidance stress at adult age. For reasons detailed in the methods section, distension sessions in the ‘diurnal variation assessment’ were carried out within a 15 day timeframe. Therefore, pre-water avoidance measurements in the maternal separation experiment were compared to measurements obtained 15 days post-water avoidance. Rats showed a significant post-stress increase in VMR to distension ( Figure 2A, mean AUC ± SEM; 71.2 ± 2.4 vs 104 ± 4.8, Wilcoxon’s test *P*=0.004, pre- vs post-stress respectively). Figure 2B depicts the same data but now evaluated on a per volume basis; pre- vs post-water avoidance comparisons indicated significantly increased response to distension at all 3 distension volumes ( *P*<0.01).

## Discussion

 Unlike earlier observations in male Lewis rats ^11^, our results indicate that male Long Evans rats do not experience daily variation of sensitivity to distension. Although EMG recordings of abdominal contractions were used as read-out in both rat studies, some differences in colonic distension methodology (volume vs isobaric distensions) are apparent and may explain the observed discrepancy. For our EMG recordings we used a radio-telemetry technique that we evaluated earlier, it allows for measurements in freely moving and non-fasted rats ^12^. Performing measurements in freely moving rats lowers the amount of stress that is usually encountered when rats are restrained ^16^ as is often the case during isobaric distensions. This is relevant because stress is a trigger for visceral hypersensitivity in patients as well as rats ^17, 18^. The downside of our approach is that freely moving rats easily expel from the colon the balloons suitable for isobaric distensions ^12^. Thus, we performed less favored volume distensions which enable the use of balloons that will stay fit in the colon during rat movement, but cannot account for possible variations of colonic tone. The latter is an important difference with the methodology used by Gschossmann *et al.* who used barostat technology to perform isobaric distensions ^11^. However, these results were obtained in Lewis rats that had to be subjected to an 18–24h fasting period prior to- as well as restraint during distensions; both fasting and restraint are known triggers for stress ^16, 19^. Moreover, compared to other rat strains Lewis rats are more stress sensitive and exhibit aberrant visceral pain sensitivity ^20^. Especially because time of day of stress exposure is known to affect response levels ^21^, it can be speculated that earlier observations in Lewis rats reflected methodology-induced rhythmicity of stress responses and accompanying changes in visceral sensitivity rather than diurnal variation in visceral sensitivity as such. Despite precautions taken, our own experiments also may have suffered from unforeseen/unnoticed methodology-induced bias affecting clock regulated mechanisms including diurnal sensitivity changes. Such bias could have been ruled out by establishing normal circadian release of an example hormone or the use of other readouts under the control of the biological clock. This however, would not have changed the end conclusion of these investigations.

Similar to the rat study discussed before, our results are also not in line with those obtained by Enck *et al.* ^3^. These authors showed a daily rhythm of rectal perception in healthy male volunteers. In rats we evaluated four time points whereas seven were evaluated in the human study. Because of this, variations observed in men may have gone unnoticed in our male Long Evans rats. Further, volunteers were asked to refrain from eating starting at noon and received their first (light) meal at 00:45. Together with possible sleep deprivation (sleeping was allowed between 01:00 and 05:30 only) and continued unnatural conditions for study subjects (rectal balloon catheter remained present during entire timeframe of the study; 26 hours), the abnormal dietary pattern may have influenced study outcome in these healthy volunteers. Importantly, it is also possible that conflicting data arise because diurnal variation in visceral perception is present in healthy men but does not occur in normal Long Evans rats or cannot be properly detected in a robust rat model. Irrespective of these considerations, we have to conclude that in the absence of a circadian rhythm of colonic sensitivity in normal controls, our rat model and methodology are not suitable to investigate disturbed circadian rhythms in relation to visceral hypersensitivity in IBS.

## Data availability

The data referenced by this article are under copyright with the following copyright statement: Copyright: © 2016 Botschuijver S et al.

Data associated with the article are available under the terms of the Creative Commons Zero "No rights reserved" data waiver (CC0 1.0 Public domain dedication). 

*F1000Research*: Dataset 1. Excel datasheet showing raw EMG data acquired during colonic distensions, 10.5256/f1000research.7238.d111689 ^22^
