# Supplementary material for: Absence of diurnal variation in visceromotor response to colorectal distention in normal Long Evans rats
Source: F1000Res. 2016 Jan 22;5:98. [Version 1] doi: 10.12688/f1000research.7238.1 (PMC4748828; doi:10.12688/f1000research.7238.1)
Supplement: Peer review history

# Peer review: Referee response for version 1

**DOI:** 10.5256/f1000research.7798.r12101

**Published:** 2016-02-15

**Authors:** Siobhain O'Mahony

**Affiliations:** 1 Department of Anatomy and Neuroscience, College of Medicine and Health, University College Cork, Cork, Ireland

Botschuijver *et al*., present a well written study that is methodologically sound. They assess the diurnal variation (4 timepoints) in visceral sensitivity in Long Evans (male) rats. They also assess the impact of water avoidance stress on visceral sensitivity in maternally separated (male) rats. 

I thought it was still worthwhile assessing if there was a diurnal variation in the MS rats. The lack of difference in the control rats should not have deterred them from doing so as there are many studies where changes/differences are seen in man that can never be replicated in rat. Also the authors might want to consider a different strain of rat as different strains may be more responsive to the CRD procedure. 

Fig 1D-no error bar on 04:00 hours?

I have read this submission. I believe that I have an appropriate level of expertise to confirm that it is of an acceptable scientific standard.

# Peer review: Referee response for version 1

**DOI:** 10.5256/f1000research.7798.r12048

**Published:** 2016-02-04

**Authors:** Eamonn Quigley

**Affiliations:** 1 Gastroenterology and Hepatology, The Methodist Hospital, Weill Cornell Medical College, Houston, TX, USA

This study is well done and the results are relatively straightforward.  It would have been nice to have control responses on the VMR plots for reference so that one could gauge the magnitude of the response to colo-rectal distension, especially, as this was a novel distension protocol. From a methodological point of view their model is of interest because of its ability to allow the animals to move freely; comparisons with restrained animal results would be interesting.

I have read this submission. I believe that I have an appropriate level of expertise to confirm that it is of an acceptable scientific standard.

# Peer review: Referee response for version 1

**DOI:** 10.5256/f1000research.7798.r12045

**Published:** 2016-01-29

**Authors:** David Grundy

**Affiliations:** 1 Department of Biomedical Science, University of Sheffield, Sheffield, UK

This is a well conducted study using appropriate methodology providing a clear cut, if negative, data on diurnal variations in visceromotor response to colonic distension. The use of the maternally separated rat model provides validation of the model but raises the question of how diurnal variations may impact on stress induced hypersensitive VMR responses.

I have read this submission. I believe that I have an appropriate level of expertise to confirm that it is of an acceptable scientific standard.
